# Effect of Electroacupuncture at Zusanli (ST36) on Intestinal Microbiota in Rats With Chronic Atrophic Gastritis

**DOI:** 10.3389/fgene.2022.824739

**Published:** 2022-02-23

**Authors:** Wanyi Huang, Yuenming Yau, Jingru Zhu, Yingjie Wang, Zhipeng Dai, Huijuan Gan, Linchao Qian, Zongbao Yang

**Affiliations:** ^1^ School of Medicine, Xiamen University, Xiamen, China; ^2^ College of Acupuncture and Moxibustion, Fujian University of Traditional Chinese Medicine, Fuzhou, China; ^3^ Physical Education College, Hunan City University, Yiyang, China

**Keywords:** electroacupuncture, chronic atrophic gastritis, intestinal microbiota, zusanli (ST36), 16s rDNA sequencing

## Abstract

**Background:** Electroacupuncture is a common treatment for chronic atrophic gastritis (CAG) in China. We aimed to determine the effects of electroacupuncture at zusanli (ST36) on intestinal microbiota in CAG rats.

**Methods:** In total, 42 SD rats were randomly divided into normal (NC, 10 rats) and model (MG, 32 rats) groups. Rats in the MG group were established as CAG disease models. After that, the rats in the MG group were randomly divided into CAG (10 rats), electroacupuncture (EA, 10 rats), and Vitacoenzyme (Vit, 10 rats) groups. Rats in the NC and CAG groups were subjected to a 30-min/d confinement for 4 weeks. Rats in the EA group were given electroacupuncture at zusanli for 30 min/d for 4 weeks. Rats in the Vit group were given Vitacoenzyme solution 10 ml/(kg d) for 4 weeks. Histopathological changes in the gastric mucosa were observed with hematoxylin and eosin staining, and the gene expression level of p53, Bcl-2, and c-myc was determined using the qPCR method. The 16S rDNA sequencing technique was used to determine structural changes and relative abundance expression of intestinal flora.

**Results:** Compared with the NC group, gastric mucosal pathology in the CAG group revealed significant inflammatory infiltration, and the gastric mucosal lesions in the electroacupuncture group were improved remarkably; the expression of p53 and c-myc genes in the CAG group increased (*p* < 0.05), while the expression of Bcl-2 genes decreased (*p* < 0.05) in the EA group, that of p53 and c-myc genes decreased (*p* < 0.05), and that of Bcl-2 genes increased (*p* < 0.05). The abundance of bacteria such as *Lactobacillus*, *Desulfobacterota*, and *Bacteroides pectinophilus group* in the CAG group increased (*p* < 0.05), while that of bacteria such as *Gastranaerophilales*, *Romboutsia*, and *Blautia* decreased (*p* < 0.05). The relative abundance of *Desulfobacterota* and *Helicobacter* in the EA group decreased (*p* < 0.05), while that of probiotic bacteria such as *Oscillospirales*, *Romboutsia*, and Christensenellaceae increased (*p* < 0.05).

**Conclusion:** Electroacupuncture at zusanli can promote the repair of pathological damage to the gastric mucosa in rats with CAG, and the mechanism might relate to the reduction in the relative abundance of harmful bacteria, increase in the relative abundance of intestinal probiotics, and regulation of the intestinal microbiota.

## Background

Although the overall incidence of gastric cancer has decreased worldwide in recent years, its public health burden remains significant (International Agency for Research on Cancer, 2020). According to a report from the World Health Organization (WHO), gastric cancer was the fourth most prevalent cancer and the fourth leading cause of cancer-related mortality in the world in 2020 (International Agency for Research on Cancer, 2020). While chronic atrophic gastritis (CAG) is epidemiologically associated with gastric cancer occurrence and is accepted as a type of precancerous lesion, CAG is also a common disease, with a lifetime prevalence of 5–10% (Lanas and Chan, 2017). From a histopathological aspect, CAG leads to a reduction in the intrinsic glands of the gastric mucosa and even fibrous replacement, intestinal epithelial metaplasia, and even atypical hyperplasia due to long-term damage to the gastric mucosa (Li et al., 2018). In addition to being a risk factor for gastric cancer, CAG is also clinically related to various disorders, including intestinal metaplasia and dysplasia (Li et al., 2018), depression (Zhao et al., 2018), and damaged skeletal health (Fisher et al., 2020). The current treatment for CAG focuses on symptomatic treatment such as triple therapy (Tarasconi et al., 2020). But, in China, clarithromycin and metronidazole have high resistance, and according to a report, omeprazole taken for a long term can cause many side effects, such as hypomagnesemia (Yoldemir et al., 2021).

In traditional Chinese medicine (TCM), CAG belongs to the following category: stuffiness of the stomach, stomach fullness, and stomach pain and noise. In TCM, electroacupuncture is often used to treat CAG and chronic diseases, improve general health, and relieve pain by electrically stimulating specific acupoints to elicit “De Qi.” Compared with acupuncture, electroacupuncture has the advantages that the level of stimulation is easy to quantify and reproduce experimentally and clinically. Accumulating evidence supports electroacupuncture as a safe and effective treatment for CAG (Zhang et al., 2016; Li et al., 2019). Interestingly, electroacupuncture has been shown to regulate intestinal microorganisms (Si et al., 2018). However, to the best of our knowledge, the effect of electroacupuncture on the intestinal microbiota of rats with CAG has barely been explored. Therefore, 16S rDNA sequencing was employed to determine the changes in the intestinal microbial community structure with or without electroacupuncture in a CAG rat model in this study. Our results provide new insights into the pathogenesis and treatment of CAG.

## Materials and Methods

### Animals and Treatment

Forty-two Sprague–Dawley (SD) rats (weight 200 ± 20 g) were obtained from the Xiamen University Laboratory Animal Center (Xiamen, China). All procedures involving animals were approved by the Animal Protection and Ethics Committee of Xiamen University (XMULAC20190142) and were performed by following the Guiding Opinions on the Good Treatment of Laboratory Animals. All rats were housed in a laboratory animal room in the School of Medicine, Xiamen University (Xiamen, China), at room temperature (22 ± 2°C) and a light/dark cycle (12 h) to simulate natural conditions, with free access to water and food. After 1 week of adaptive feeding, the rats were randomly divided into NC (n = 10) and MG (n = 32) groups. The MG group was subjected to a compound method for disease modeling for 10 weeks: drinking water was replaced by 0.1% ammonia solution (Shanghai BaiShun Biotechnology Co., Ltd., China) on the first day and by 20 mmol/L of sodium deoxycholate solution (Beijing AuBoXing Biotechnology Co., Ltd., China) on the second day. The rats drank the solutions on alternate days until the model was established. Moreover, the rats were kept in an angry and combative state for 1 h/d by tail clamping and subjected to the hunger and satiety method—2 days of overfeeding and 1 day of fasting, which was repeated for a total of 10 weeks. At the end of the 10th week of modeling, spare MG group rats were anesthetized and sacrificed. Then the stomach was removed by dissection, and the gastric mucosal tissues of the rats were observed visually for ischemia, thinning, lack of elasticity, and shallow and flat mucosal folds. The whole stomach was taken for HE staining, and the changes in gastric mucosal gland atrophy, inflammatory cell infiltration, cell necrosis, intestinal epithelial metaplasia, and heterotypic hyperplasia were visualized under a light microscope. Then successful replication of the CAG model was carried out. Subsequently, MG group rats were randomly divided into CAG, EA, and Vit groups, with 10 rats in each group. The rats in the EA group were fixed in an elastic cloth sleeve. The first needle was inserted at zusanli with a HanYi Acupuncture Needle, 0.18 × 13 mm (Beijing HanYi Medical Equipment Co., Ltd., China), perpendicular to the skin to a depth of about 3–5 mm, and the second needle was inserted 5 mm below the zusanli point at an angle of 45°, with the needle tip facing zusanli, to a depth of 3–5 mm. The two needles were connected with a G6805-I Electroacupuncture Stimulator (Qingdao XinSheng Industrial Co., Ltd., China) for 30 min, alternating between the left and right side acupoints. Output parameters were sparse and dense waves (sparse wave 4 Hz, dense wave 50 Hz) and voltage (2–4 V). The intensity valve on the apparatus was adjusted to the first peak, that is, 50 mA (±25%), while electroacupuncture was performed on each rat. Electroacupuncture was performed continuously for 4 weeks. The NC and CAG groups were given the same fixation as the EA group. The Vit group was given 10 ml of Vitacoenzyme suspension by gavage for 4 weeks once daily, at a concentration of 24 mg/ml (LePuHengJiuYuan Pharmaceutical Co., Ltd., China).

### Rat Weight and Sample Collection

The rats’ body mass was monitored after adaptive feeding. After the end of the intervention, following fasting without water for 1 day, rats were decapitated: a researcher exposed the rat’s neck, and the assistant cut the rat’s head off using scissors. The stomach tissues and feces were collected immediately, and the fecal matter was stored at -80°C for 16S rRNA sequencing. The stomach tissues were operated on at low temperature; they were divided into two parts: one part was immersed in paraformaldehyde (Servicebio Co., Ltd., China) for histological evaluation, and the other part was stored at −80°C for RT-PCR.

### Histomorphology of Gastric Mucosa Observed by HE Staining

The gastric mucosal tissues were drenched with paraformaldehyde for 1 day, dried using gradient ethanol, routinely embedded with paraffin, segmented using a microtome (Leica Co., Ltd., Germany) at a thickness of 4 μm, dewaxed with xylene, and then stained with hematoxylin and eosin to observe the morphological changes in gastric mucosal tissues under an ortho-fluorescence microscope (Carl Zeiss Co., Ltd., Germany), and images were collected.

### P53, Bcl-2, and c-myc Gene Expression in rat Gastric Mucosal Tissues by RT-PCR

In total, 200 mg of gastric mucosa tissues were taken. The total RNA of the gastric mucosa was extracted by using a TRIzol kit and reverse-transcribed into cDNA, and then the reaction system was placed in a 7900HT Fast Real-Time PCR System (Applied Biosystems Co., Ltd., United States) for qPCR. After the reaction, the CT value of the target gene and internal reference gene of each sample were determined using the 2^−ΔΔCT^ method to evaluate the relative expression levels of four mRNAs (p53, Bcl-2, c-myc, and β-actin). The primer sequences used to amplify the specific genes are given in Table 1.

**TABLE 1:** Gene-specific primers.

Rat gene	Primer sequences (5–3′)
β-Actin	F:5′-CTGGCTCCTAGCACCATGAA-3′
	R:5′- AAA​ACG​CAG​CTC​AGT​AAC​AGT​C-3′
p53	F:5′- GCG​TTG​CTC​TGA​TGG​TGA-3′
	R:5′- CAG​CGT​GAT​GAT​GGT​AAG​GA-3′
Bcl-2	F:5′- ATG​GCG​CAA​GCC​GGG​AGA​ACA​GGG​T-3′
	R:5′- ACC​CTG​TTC​TCC​CGG​CTT​GCG​CCA​T-3′
c-myc	F:5′- TCT​CCG​TCC​TAT​GTT​GCG-3′
	R:5′- GGC​TGG​TGC​TGT​CTT​TGC-3′

### Fecal Microbiota in Various Groups of Rats by 16S rDNA Sequencing

Fecal samples from each group of rats were subjected to total DNA extraction using the QIAamp Fast DNA Stool Mini Kit (Qiagen Co., Ltd., Germany) according to the manufacturer’s instructions. Sample DNA quality testing was performed to ensure the quality of sequencing for subsequent library construction, which includes concentration, purity, and integrity. PCR amplification was performed on the V3–V4 regions of the 16S rRNA gene of the sample bacteria. The PCR products were analyzed by 1.5% agarose gel electrophoresis and purified and eluted by using a PCR Cleanup Kit (AxyPrep Co., Ltd., United States), and the concentrations were determined by a Qubit 3.0 fluorometer (Thermo Fisher Scientific Co., Ltd., United States). Equal proportions of each sample were taken and mixed together to form a template, and then a second PCR was performed with the addition of a library index sequence and a splice sequence, required for Illumina sequencing. After library QC purification, the samples were tested and precisely quantified for insert fragments and library molar concentrations using a 2,100 Bioanalyzer (Agilent Technologies Inc., United States) and a 7,300 Plus Real-Time PCR System (Applied Biosystems Co., Ltd., United States), respectively. The libraries that passed the quality check were subjected to 250 bp paired-end sequencing on a HiSeq 2,500 System (Illumina Co., Ltd., United States) according to the standard procedure.

### Statistical Analysis

Data on RT-PCR and body weight were statistically analyzed using IBM SPSS 23.0 (IBM Co., Ltd., United States). Measures conforming to normal distribution were statistically described using mean ± standard deviation $\left(\bar{x}\pm s\right)$ . Independent samples *t*-test and ANOVA were used for comparison between groups. (LSD-*t* test was further selected for two-way comparison if the ANOVA results were statistically significant; Dunnett’s T3 test was selected for two-way comparison if the variance was not equal.) The hypothesis test was set to be statistically significant at *p* < 0.05, and the data were plotted using GraphPad Prism 8.0 (GraphPad Software Co., Ltd., United States) software.

The raw data obtained from the sequencing of colony genes were sorted, filtered, and quality-assessed to remove chimeras; then OTUs were clustered based on the valid data. After the completion of sequencing, based on the OTU clustering, on the one hand, species annotation was first carried out to annotate the representative sequences of each OTU to obtain the corresponding species information and abundance distribution. Statistical analysis methods such as LEfSe were selected to test the differences in microbial species composition and microbial community results of the samples to further explore the differences in the microbial community structure between groups. The fecal gene sequencing was provided by Xiamen ChengGe Biotechnology Co., Ltd.; the bioinformatics analysis process was completed on the BioInfo Cloud Platform (https://cloud.majorbio.com/); and statistical analysis was performed according to the data and images generated by the BioInfo Cloud Platform.

## Results

### Weight Changes

**FIGURE 1:**
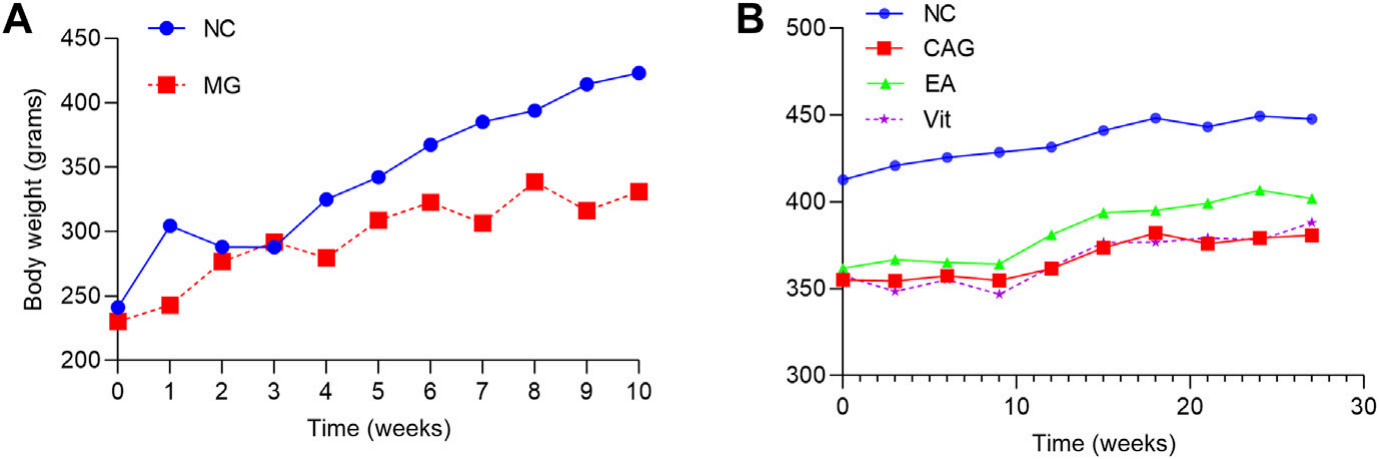
Changes in body weight of rats in each group. **(A)** Change in body weight of the two groups of rats during the modeling period. **(B)** Change in body weight of each group of rats during the intervention phase.

During the CAG model establishing period, the weight of rats in the NC group was stable and greater, while that of rats in the MG group was smaller; at the end of the 10th week of modeling, the weight of rats in the MG group was significantly lower than that of the NC group, and the difference was statistically significant (*p* < 0.05), as shown in Figure 1A. After successful modeling, the experiment entered the intervention phase, and the weight of rats in all groups showed an increasing trend. In comparison, the body weight level of rats in the NC group was significantly higher than that of the remaining groups. The increase in the body weight of rats in the EA and Vit groups was similar, and the level was also comparable. The increase in the body weight of rats in the CAG group was smaller and even occasionally decreased; however, the difference in the body weight of rats in each group was not statistically significant, as shown in Figure 1B.

### Macroscopic Changes and Pathological Changes

**FIGURE 2:**
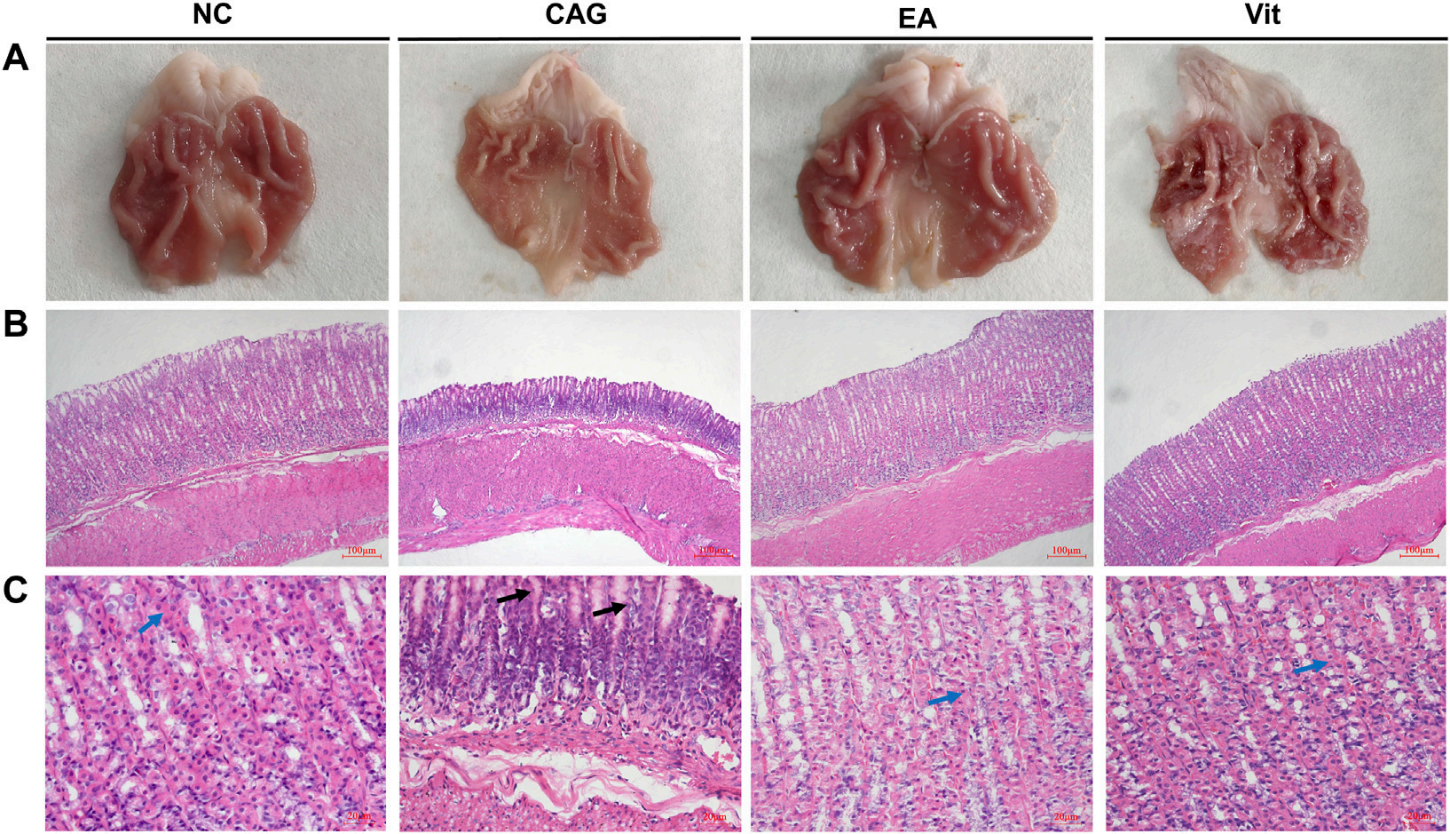
Histomorphological observation of the gastric mucosa in each group of rats. **(A)** Observation of the gastric mucosa in each group of rats with naked eyes. **(B)** Observation of the gastric mucosa in each group under a fluorescence microscope at 100×. **(C)** Observation of the gastric mucosa in each group under a fluorescence microscope at 400×. Blue arrows show parietal cells, and black arrows show plasma cells and lymphocytes.

As shown in Figure 2, the structure of gastric mucosal glands was intact in the NC group, with neat and tight cell arrangement, no hemorrhage in the interstitium, and no inflammatory cell infiltration; in the CAG group, the mucosal layer was atrophied, the chief cells and parietal cells were lost in large numbers, the volume of the glandular lumen was widened, a large number of lymphocytes and plasma cells were infiltrated, some neutrophils were observed, and bleeding in the interstitium was obvious, suggesting that these rats exhibited typical characteristics of a CAG disease model; in the EA group, atrophy of the intrinsic glands of gastric mucosal tissues was improved to some extent, the cells were arranged more neatly, the chief cells and parietal cells increased in number, and the volume of the glandular lumen decreased, indicating a remission of CAG signs; and in the Vit group, atrophy of the intrinsic glands of gastric mucosal tissues slightly improved compared with the CAG group, with neater cell arrangement, increased main and wall cells, larger glandular lumen volume, reduced inflammatory cell infiltration, and visible congestion and edema in the intercellular space. Under visual observation, rats in the NC group had normal gastric mucosal tissue morphology: smooth mucosal surface, red color, intact mucosal folds, and moderate thickness and elasticity; in the CAG group, the gastric mucosa was observed to be thinner: the mucosal folds were reduced and flattened, the tissue lacked elasticity, and the mucosa was paler in color; and gastric mucosal tissues of rats in the Vit group showed a less smooth surface, slightly shallow folds, less elasticity, and no ischemic, hemorrhagic, or ulcerative changes in the mucosa.

### Expression Changes of p53, Bcl-2, and c-myc Genes

**FIGURE 3:**
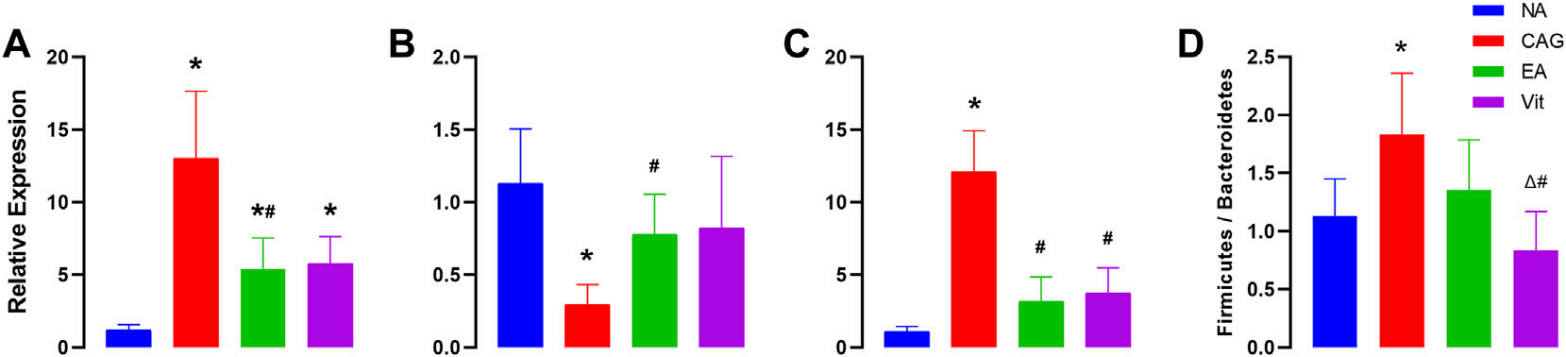
Expression of p53, Bcl-2, and c-myc genes in the gastric mucosa of rats. ^*^ *p* < 0.05 *vs*. NC, ^#^ *p* < 0.05 *vs* CAG, and ^Δ^ *P* < 0.05 *vs* EA. **(A)** Differences in the expression levels of p53 in rat gastric mucosa. **(B)** Differences between groups in the expression of Bcl-2 in rat gastric mucosa. **(C)** Differences in the expression levels of c-myc in rat gastric mucosa. **(D)** Differences in F/B ratios between groups of rats.

As shown in Figure 3, the expression of p53 and c-myc genes significantly increased in the CAG group compared with that in the NC group, while that of Bcl-2 genes decreased, that of p53 was also upregulated in the EA and Vit groups, that of c-myc increased but was not significantly different, and that of Bcl-2 genes decreased but was not significantly different. Compared with the CAG group, the expression of p53 and c-myc genes was significantly downregulated in the EA group, while that of Bcl-2 genes increased, that of p53 genes in the Vit group decreased, that of Bcl-2 genes showed an increasing trend, and that of c-myc genes decreased. There was no significant difference in gene expression between the EA and Vit groups.

### α-Diversity and Rarefaction Analysis

**FIGURE 4:**
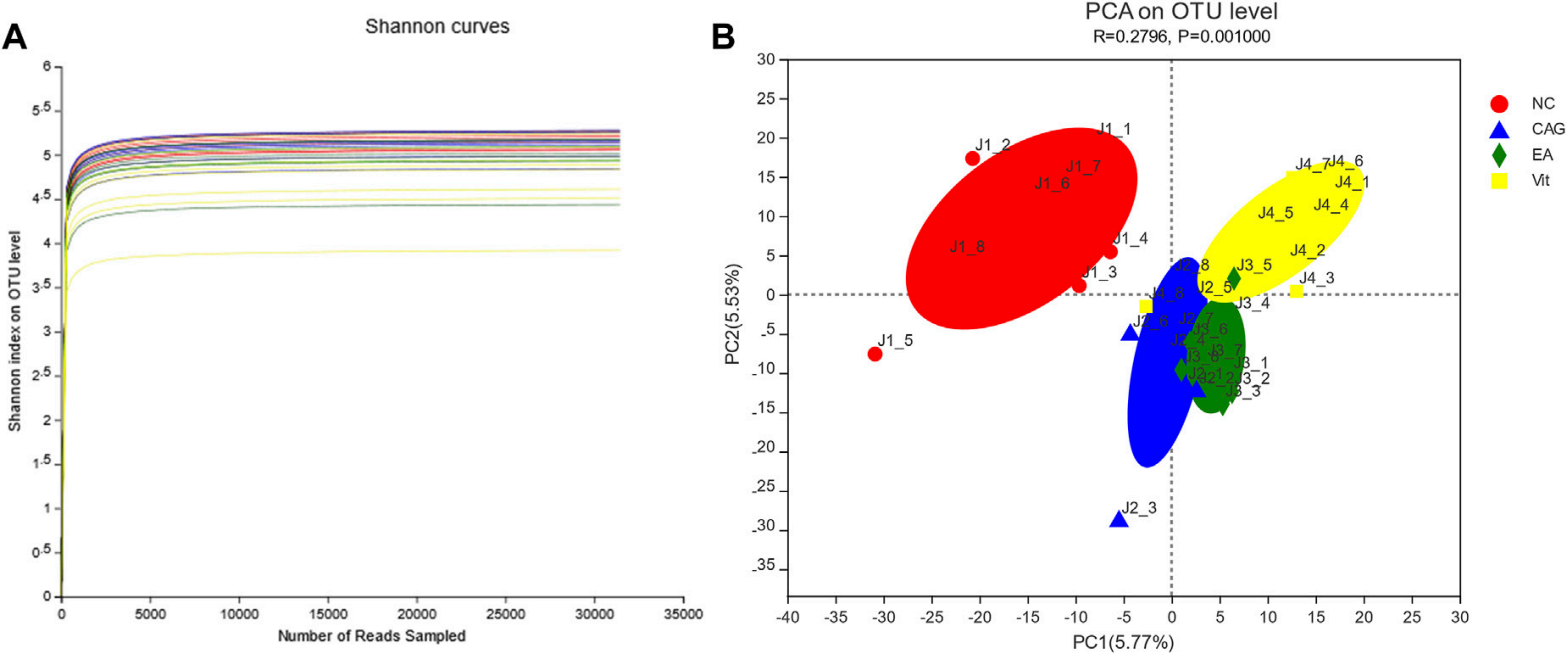
Dilution curve. **(A)** Shannon index of rat intestinal flora. **(B)** PCA of rat intestinal flora.

As shown in Figure 4A, the Shannon curves are labeled with different colors for rats in each group, where the horizontal coordinate represents the amount of randomly selected sequencing data; the vertical coordinate represents the diversity index, that is, the Shannon index. The Shannon index is one of the α-diversity indices that reflect the richness and evenness of the sample species in a comprehensive manner; the higher the value of the Shannon index, the higher the species diversity of the sample. The smoother the dilution curve of the sample, the more reliable the sequencing depth of the sample. The OTUs for each group in the graph do not continue to increase with increasing sequencing, and the flat curve indicates that the vast majority of microbial diversity information in all rat fecal samples can be reflected in this sequencing.

### Principal Component Analysis

PCA revealed significant differences between the four groups. The more similar the microbiota composition between the samples represented the closer they were to the coordinate points in the PCA plot. The contribution of PC1 and PC2 in Figure 4B was 5.53 and 5.77%, respectively, with a cumulative contribution of 11.30%. The closer the PCA plots in the principal components of the distribution in Figure 4B, the more similar the community composition of the samples. On the PC1 axis, all three groups, except the NC group, showed positive distribution, but the composition of the respective flora of the four groups differed (Figure 4B).

### Firmicutes/Bacteroidetes Ratio

We observed a significant increase in the F/B ratio after CAG in rats, and a downward trend after receiving electroacupuncture intervention, but the downward trend did not show a statistical difference, while a significant difference was observed in the Vit group compared with the CAG group (Figure 3D).

### Non-Metric Multidimensional Scaling Analysis

**FIGURE 5:**
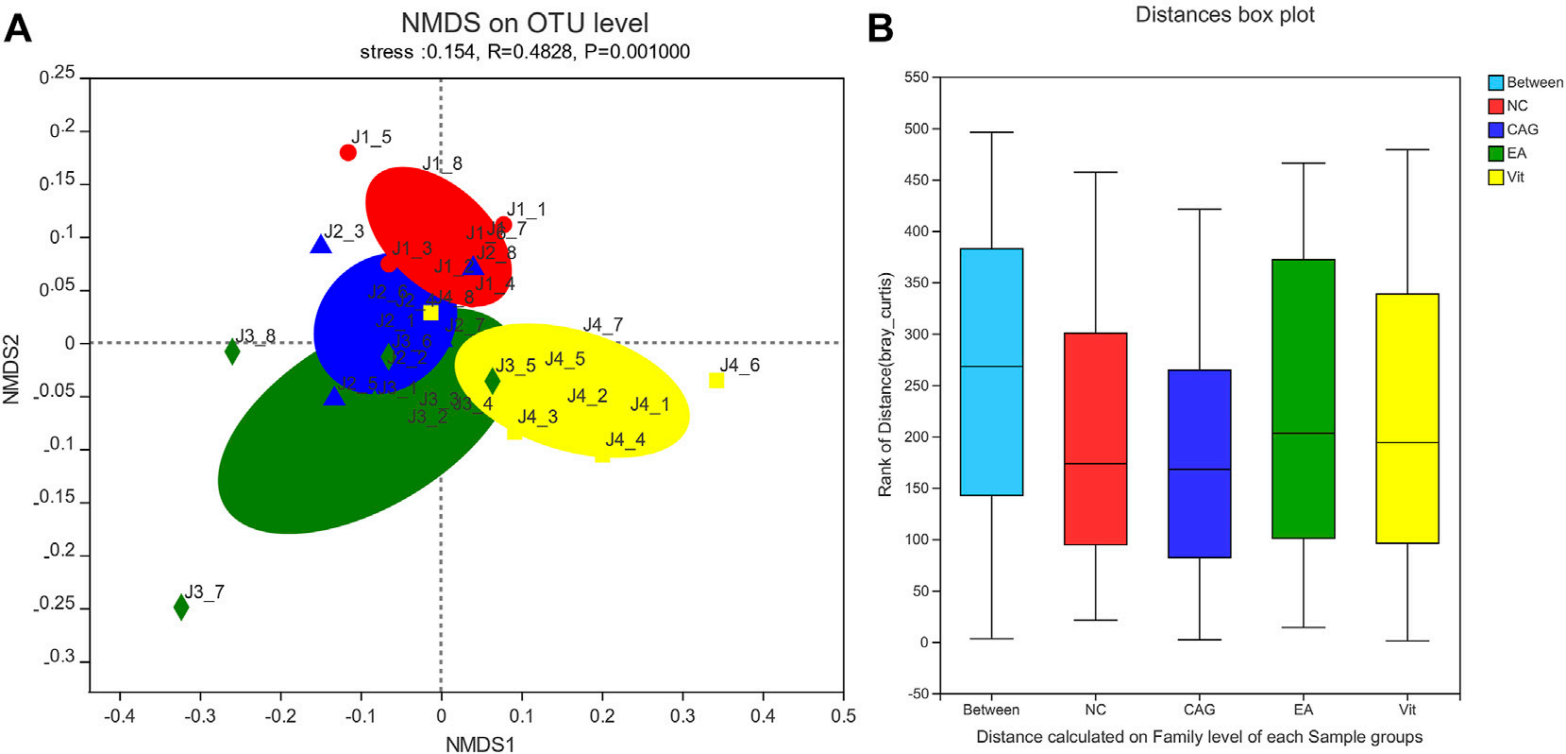
NMDS scatterplot and box plot of intergroup distances. **(A)** NMDS scatterplot; each group of samples is represented by different colors and shapes of dots; the closer the dots are, the more similar the samples are. **(B)** Box plot of distance between groups; the “between” boxes represent the differences between groups, and the others represent the differences within the respective groups.

As shown in Figure 5, non-metric multidimensional scaling (NMDS) analysis of the bacterial community composition of samples in all groups based on the Bray–Curtis distance showed significant differences between the groups. There was a tendency for the EA group to separate from the CAG group, but no statistical difference has yet emerged.

### LEfSe Analysis

**FIGURE 6:**
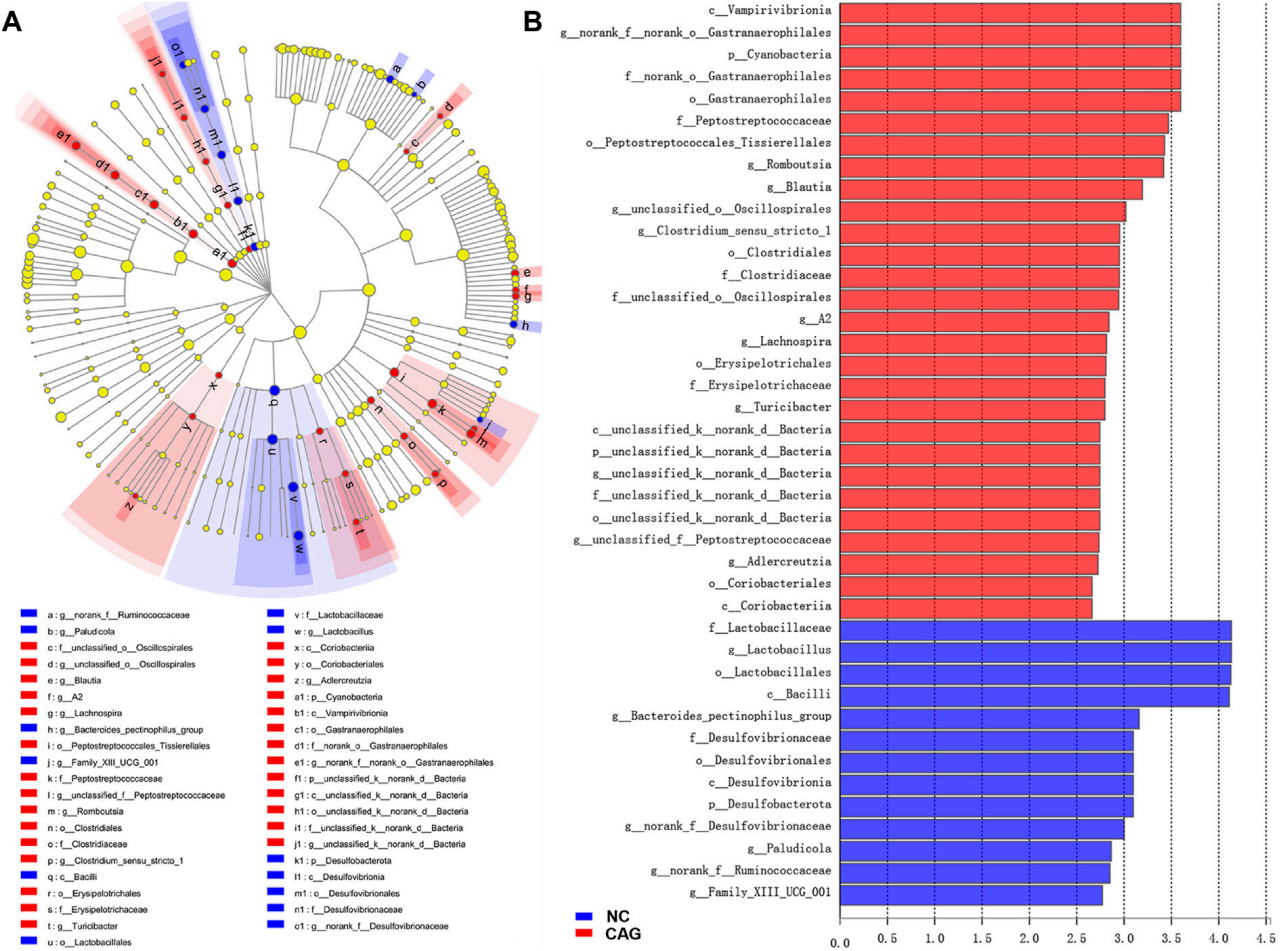
LEfSe analysis of rat intestinal microbiota in NC and CAG groups. **(A)** LEfSe multilevel species tree diagram with inside-out circle nodes representing taxonomic levels from the phylum level to genus level; each small circle represents a taxon under that taxonomic level. The light yellow circle nodes indicate microbial taxa that are not significantly different in any of the different groupings. The red and blue circle nodes indicate microbial taxa that were significantly enriched in the NC and CAG groups, respectively, and had a significant effect on the difference between groups. **(B)** LDA discriminant bar graph of LDA scores obtained by LDA analysis, with larger LDA scores representing a greater effect of species abundance on the difference effect.

The LEfSe analysis program was used to count species information for each group of rat colonies, and the linear discriminant analysis (LDA) threshold was set to 2. Species with LDA values above 2 were considered to have statistically significant differences, and species with significant differences in abundance between groups were colored with different colors. The analysis results are presented in Figure 6 as LEfSe multilevel species hierarchical tree plots and LDA discriminant bar charts of the intestinal microbiota of the normal group compared with the CAG group. From the abundance point of view, compared to the normal group, the intestinal microbiota of the CAG group containing Lactobacillaceae, *Lactobacillus*, Lactobacillales, Bacilli, *Bacteroides pectinophilus* group, Desulfovibrionaceae, Desulfovibrionales, Desulfovibrionia, Desulfobacterota, *Paludicola*, Ruminococcaceae, and *Family_XIII_UCG_001* had relatively higher abundance of bacteria with statistically significant differences (*p* < 0.05). Compared with the NC group, the CAG group had higher abundance of Vampirivibrionia, Gastranaerophilales, Cyanobacteria, Peptostreptococcaceae, *o__Peptostreptococcales-Tissierellales*, *Romboutsia*, *Blautia*, Oscillospirales, *g_Clostridium_sensu_stricto_1*, Clostridiales, Clostridiaceae, *f_unclassified_o__Oscillospirales*, *g_A2*, *Lachnospira*, *Erysipelotrichales*, Erysipelotrichaceae, *g_Turicibacter*, *g_unclassified_f__*Peptostreptococcaceae, *Adlercreutzia*, Coriobacteriales, and Coriobacteriia. The abundance of these bacteria was relatively low, and the difference was statistically significant (*p* < 0.05).

**FIGURE 7:**
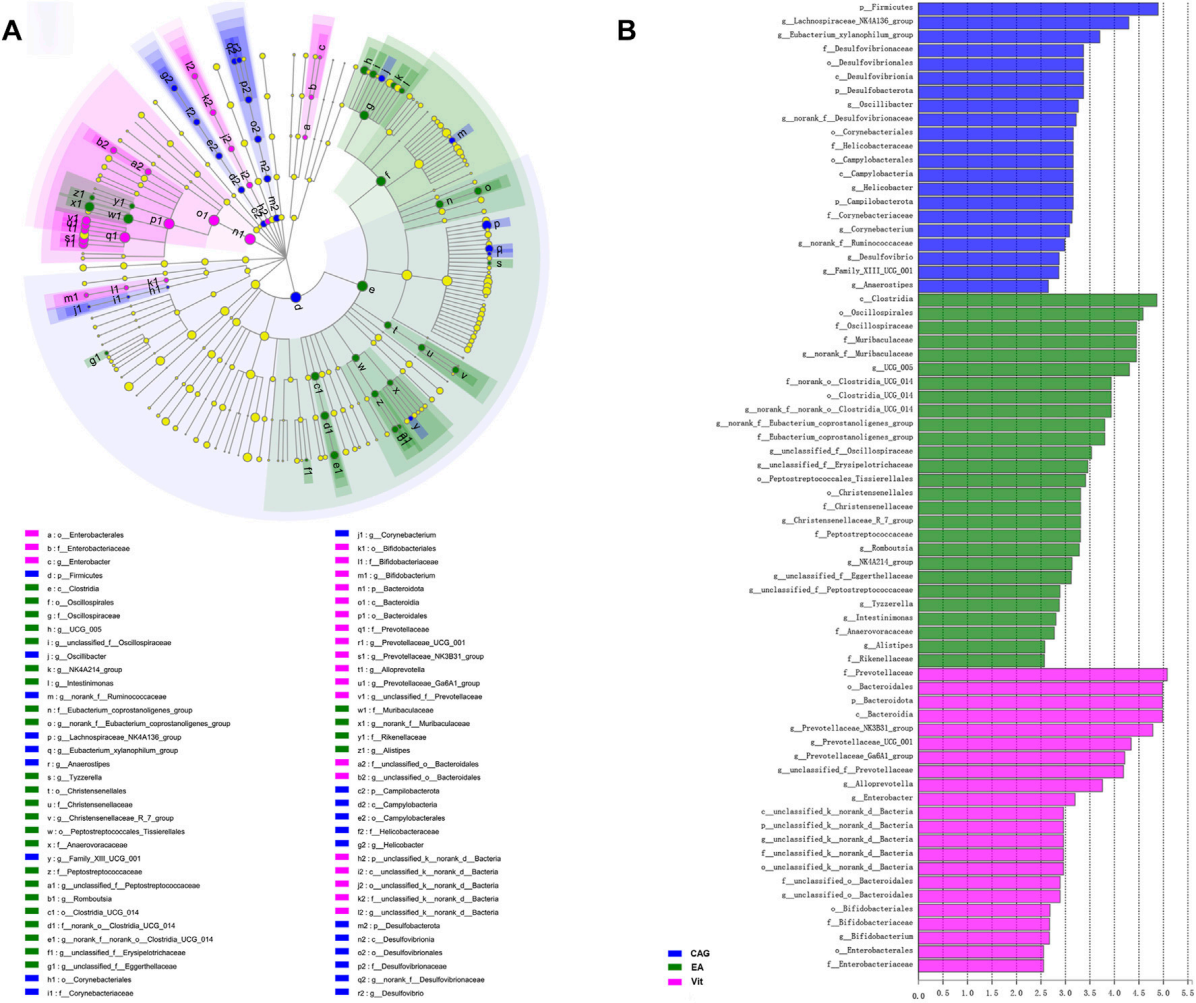
LEfSe analysis of rat intestinal microbiota in the CAG, EA, and Vit groups. **(A)** LEfSe multilevel species tree diagram. **(B)** LDA discriminant bar chart.

Figure 7 shows the LEfSe multilevel species hierarchical tree and LDA discriminant bar chart of the intestinal microbiota compared between the CAG, EA, and Vit groups. Compared with the EA and Vit groups, CAG group intestines contained Firmicutes, Lachnospiraceae NK4A136 group, *g_Eubacterium_xylanophilum* group, *f_*Desulfovibrionaceae, *o_Desulfovibrionales*, *c_Desulfovibrionia*, *p_Desulfobacterota*, *g_Oscillibacter*, *g_norank_f_*Desulfovibrionaceae, *o_Corynebacteriales*, *f_*Helicobacteraceae, *o_Campylobacterales*, *c_Campylobacteria*, *g_Helicobacter*, *p_Campilobacterota*, *p_Campilobacterota*, *f_*Corynebacteriaceae, *g_Corynebacterium*, *g_norank_f_*Ruminococcaceae, *g_Desulfovibrio*, *g_Family_XIII_UCG_001*, and *g_Anaerostipes* in higher abundance. Compared with the CAG and Vit groups, the intestinal microbiota of rats in the EA group had higher abundance of *c_Clostridia*, *o_Oscillospirales*, *f_*Oscillospiraceae, *f_*Muribaculaceae, *g_norank_f_*Muribaculaceae, *g_UCG_005*, *f_ norank_o_Clostridia_UCG_014*, *o_Clostridia_UCG_014*, *g_norank_f_norank_o_Clostridia_UCG_014*, *g_norank_f_Eubacterium_coprostanoligenes_group*, *f_Eubacterium_coprostanoligenes_group*, *g_unclassified_f_*Oscillospiraceae, *g_unclassified_f_*Erysipelotrichaceae, *o_Peptostreptococcales_Tissierellales*, *o_Christensenellales*, *f_*Christensenellaceae, *g_*Christensenellaceae*_R_7_group*, *f_*Peptostreptococcaceae, *g_Romboutsia*, *g_NK4A214_group*, *g_unclassified_f_*Eggerthellaceae, *g_unclassified_f_*Peptostreptococcaceae, *g_Tyzzerella*, *g_Intestinimonas*, *f_*Anaerovoracaceae, *g_Alistipes*, and *f_*Rikenellaceae (*p* < 0.05).

## Discussion

CAG is considered to be a precancerous state of the stomach (Bockerstett et al., 2020). In the early stage, damage to the gastric mucosa mainly causes an acute inflammatory response, and chronic gastritis can be formed by the persistence of the damaging factors, and the degree of lesion ranges from mild to severe, from non-atrophic to atrophic progressive development.

When CAG occurs in rats under stress, the apoptotic process of gastric mucosal epithelial cells is accelerated and the normal life span of cells is shortened. In order to maintain homeostasis, cells compensate for proliferation and promote cell renewal, and if there is disruption in the regulation of gastric mucosal epithelial cells, they proliferate abnormally, thus leading to intestinal epithelial hyperplasia or heterotypic hyperplasia and even tumor formation.

In rats, p53 is activated and accumulates in cells in response to stress signals from various factors. Activated p53 binds to p53 response elements (p53 Res) in target genes and transcriptionally regulates their expression, which in turn selectively blocks the cell cycle, promotes DNA repair, programmed death, or the senescence process to maintain genomic normalcy and inhibit tumor cell proliferation (Gupta et al., 2019). p53 undergoes mutations, its pro-apoptotic and DNA repair functions are lost, and cells proliferate abnormally, leading to tumorigenesis. Long-term *H. pylori* infection may also induce mutations in p53 which inactivate its apoptosis-inhibiting role, another trigger for carcinogenesis. The Bcl-2 family, on the other hand, is part of the core mechanism of apoptosis, and the anti-apoptotic properties of Bcl-2 protect cells from various damaging factors (Schenk et al., 2017). The myc gene family mainly comprises C-myc, N-myc, and L-myc—three highly related nuclear phosphorylated proteins, and the literature suggests that activation of the myc genes may affect cell function, inhibit apoptosis, promote abnormal cell proliferation, and lead to tumorigenesis through multiple pathways (Ba et al., 2018; Tong-peng et al., 2019; Anauate et al., 2020). c-myc genes are indicators for the evaluation of the effect of the GC disease process and treatment, and therefore, they are the most frequently studied indicators in studies on GC (Won et al., 2019). Several studies have found that high levels of c-myc expression were observed in gastric cancer cell lines and gastric cancer tissues (Jersey et al., 2018; Taniguchi et al., 2018).

We measured the expression levels of p53, Bcl-2, and c-myc in gastric mucosal tissues by RT-PCR to evaluate the apoptosis of gastric mucosal epithelial cells in rats. p53 and c-myc expression in the CAG group increased, while the Bcl-2 level decreased, suggesting that the proliferation and apoptosis of gastric mucosal epithelial cells were dysfunctional, and the CAG disease model was replicated successfully. The expression levels of p53, c-myc, and Bcl-2 in the gastric mucosa of the EA group were not significantly different from those of the NC and Vit groups, suggesting that electroacupuncture can regulate the expression levels of p53, c-myc, and Bcl-2 in the gastric mucosa. This indicates that electroacupuncture at zusanli can regulate the expression of p53, Bcl-2, and c-myc in gastric mucosa and contribute to the repair of gastric mucosal tissues in CAG rats.

Studies have shown that the imbalance of gastrointestinal microbiota is an important pathological link in the pathogenesis of CAG. The gut microbiome plays an important role in processes such as digestion and absorption, immune regulation, metabolism, resistance to pathogens, and maintenance of the intestinal epithelial barrier in the host. The structural characteristics of the intestinal microbiota differ significantly between healthy populations and gastric cancer (GC) patients, with a relative enrichment of conditionally pathogenic bacteria in the gut of GC patients and a lower number of probiotic bacteria (Zhang et al., 2021). Significant differences in intestinal microbiota diversity and microbiota structure were observed between *H. pylori* infection-related and non*-H. pylori*-infected subjects, and among *H. pylori*-positive patients with severe CAG. The abundance of four bacterial species, Bacillariophyceae, *Lactobacillus*, Streptococcaceae, and *Streptococcus*, was significantly higher than in subjects without atrophic gastritis (Iino et al., 2019). Yu *et al.* examined compound factor-induced CAG rats, and the results of the study of fecal microbiota showed that during the development of the disease in the gastric mucosa from normal tissue damage to GC, the intestinal microbiota changed significantly, with an increase in the abundance of the microbiota, a trend toward a decrease in diversity, a decrease in the proportion of butyrate-producing bacteria, and a predominance of harmful bacteria such as *Shigella* in the intestine, and different characteristics of the intestinal microbiota at each stage of the disease (Yu et al., 2020). On the other hand, the *Lactobacillus* family and other bacteria as probiotics are also defined in different situations, where bacteria favorable to healthy people may become opportunistic pathogens with serious repercussions for hosts with disorganized gut microbiota. More than 200 cases of *Lactobacillus* spp.-associated infections (Cannon et al., 2005) have been reported in patients with ulcerative colitis, short bowel syndrome, cancer, etc. The gastrointestinal microbiota is a very complex ecosystem with intricate microbe–microbe interactions and microbe–host interactions within the microecosystem, and the role of microecology in disease development is not yet clearly explained.

The results of the comparison between the NC and CAG groups by LEFse analysis showed that the occurrence of CAG was accompanied by disturbances in the intestinal microecosystem. The relative abundance of 28 species in the gut microbiota was reduced in the CAG group compared to the NC group, including *Gastranaerophilales*, Peptostreptococcaceae, *Romboutsia*, *Blautia*, *Oscillospirales*, Clostridiales, and *Lachnospira*. *Gastranaerophilales* is a potential probiotic of the intestinal tract, is involved in the digestion and absorption of many sugars, produces butyrate, and has anti-inflammatory and immunomodulatory effects (Li et al., 2020). *Romboutsia* belongs to the family of Peptostreptococcaceae and has a role in the production of short-chain fatty acids (SCFAs) (Qin et al., 2021), which have a positive effect on intestinal epithelial barrier function and have immunomodulatory effects. *Blautia* belongs to the family of Firmicutes and Lachnospiraceae and is widely found in the feces and intestines of mammals, with potential probiotic properties such as inhibiting inflammation and promoting SCFA production to maintain homeostatic activity in the intestine (Liu et al., 2021). Chen *et al.* observed that the relative abundance of *Blautia* significantly decreased in the structure of intestinal mucosal microbiota of colon cancer patients (Chen et al., 2012). In addition, in a mouse model of ulcerative colitis (UC), a decrease in the abundance of *Blautia* and *Lactobacillus* was observed with increased induction of dextran sodium sulfate (DSS), whereas chitosan intervention promoted the enrichment of *Blautia* and *Lactobacillus* and improved the intestinal barrier function in UC mice (Wang et al., 2019). The occurrence of CAG may be associated with a probiotic reduced abundance. Meanwhile, some studies showed the relative abundance of some harmful bacteria, such as *p_Desulfobacterot*, c_Desulfovibrionia, o_Desulfovibrionales, and *f_*Desulfovibrionaceae. The *Desulfovibrio* family, one of the major members of sulfate-reducing bacteria in the human intestinal microbiota, increased in the gut of the CAG group. The biological effects of desulfurizing *Desulfobacterota* are mainly reduction in sulfate, production of toxic hydrogen sulfide (H_2_S), reduction of disulfide bonds in the intestinal mucus layer, and breaking of the intestinal mucus barrier, leading to epithelial exposure to bacteria and other pathological factors that cause inflammation (Chen et al., 2019). In addition, an increased relative abundance of the *Lactobacillus* family was observed in the CAG group, which is consistent with previous findings that *Lactobacillus* increased in abundance in GC progression and positively correlated with disease severity. The bacteria beneficial to healthy individuals might potentially cause an opportunistic infection with lethal consequences for hosts with disrupted gut microbiota. Probiotic *Lactobacillus* have also been shown to have a risk of infection and even sepsis in immune-compromised individuals (Won et al., 2019). Enrichment of *Lactobacillus* was found in the gastric mucosa of patients with GC (Eun et al., 2014; Wang et al., 2016), and *Lactobacillus* proliferated and moved down to the intestine, suggesting its association with gastric carcinogenesis. Although the Lactobacillaceae family is regarded as probiotic and plays a role in preventing pathogenic infections, reducing inflammation, and regulating the microbiota, *Lactobacillus* may also induce inflammatory damage to epithelial cells (Lukic et al., 2013). Elevated levels of *Lactobacillus* may be associated with increased inflammation and harmful bacterial infections due to CAG.

The comparison results between the CAG, EA, and Vit groups by LEFse analysis showed that the relative abundance of harmful bacteria such as *Desulfobacterota*, *Desulfovibrionia*, Desulfovibrionales, Desulfovibrionaceae, *Campylobacter*, *o_Campylobacterales*, Helicobacteraceae, and *H. pylori* in the intestinal microbiota of the EA group was reduced compared with that of the CAG group, indicating that electroacupuncture treatment and Vitacoenzyme treatment effectively inhibited these harmful bacteria from multiplying. It is well known that *H. pylori* is a damaging factor in peptic ulcer, acute and chronic gastritis, GC, and other gastric mucosal damage diseases. Enterohepatic *Helicobacter* species (EHS) colonizes the intestine, biliary tract, and liver of humans, mammals, birds, and fish (Smet and Menard, 2020). EHS is considered a potential pathogen of inflammatory bowel disease (IBD), and several studies have revealed that high expression of EHS was detected in stool samples and intestinal biopsy tissues from patients with Crohn’s disease (Man et al., 2008; Kaakoush et al., 2010). EHS not only has pro-inflammatory activity but also causes DNA damage and stimulates increased expression of cytokines such as IL-22, IL-17a, IFN-γ, TNF-α, IL-6, and iNOS (Zhu and Zhu, 2018). Compared to the CAG and Vit groups, the samples from the EA group were enriched in beneficial bacteria such as *Oscillosporia*, *Romboutsia*, and Christensenellaceae of the intestinal microbiota, and the Christensenellaceae family has been found in human feces, colonic mucosa, the ileum, and the appendix, a new branch of Firmicutes that plays an important role in human health (Waters and Ley, 2019).

## Conclusion

It is suggested that electroacupuncture of the zusanli acupoint can impact the intestinal microbiota and promote repair of the gastric mucosa.

## Limitations and Prospects

We performed electroacupuncture intervention in rats with gastrointestinal ulcers and found that 4 weeks of electroacupuncture stimulation induced many metabolic differences systemically in rats. In the present study, we intentionally observed the effects of electroacupuncture on gastrointestinal microorganisms and hypothesized that electroacupuncture might induce changes in the microbiota that are closely related to metabolic differences in rats, so we set the duration of the intervention at 4 weeks. However, the current study found, for example, that the electroacupuncture group in PCA with the F/B ratio manifested changes but did not show statistical differences, and the microbial environment seems to change more slowly than we thought, suggesting that perhaps 4 weeks may not be long enough, and we may consider extending the intervention time in the next experiment.

The current experiment is only a static observation to study the effect of electrodes on the intestinal flora of rats, and further experiments are needed to observe the changes in the colony dynamically, which can help us understand the mechanism of the effect of electroacupuncture on the intestinal microbiota.

This research has a wide range of promising applications. The gastrointestinal flora is a complex system, and electroacupuncture as a traditional therapy without side effects will become a great complementary alternative treatment option for many diseases if we can artificially and precisely regulate the state of the gastrointestinal flora through electroacupuncture.

## Data Availability

The datasets presented in this study can be found in online repositories. The names of the repository/repositories and accession number(s) can be found below: NCBI, accession number PRJNA798563.

## References

[B1] Anauate A. C. Leal M. F. Calcagno D. Q. Gigek C. O. Karia B. T. R. Wisnieski F. (2020). The Complex Network between MYC Oncogene and microRNAs in Gastric Cancer: An Overview. Ijms 21 (5), 1782. 10.3390/ijms21051782 PMC7084225 32150871

[B2] Ba M. Long H. Yan Z. Wang S. Wu Y. Tu Y. (2018). BRD4 Promotes Gastric Cancer Progression through the Transcriptional and Epigenetic Regulation of c‐MYC. J. Cel. Biochem. 119 (1), 973–982. 10.1002/jcb.26264 28681984

[B3] Bockerstett K. A. Lewis S. A. Noto C. N. Ford E. L. Saenz J. B. Jackson N. M. (2020). Single-Cell Transcriptional Analyses Identify Lineage-specific Epithelial Responses to Inflammation and Metaplastic Development in the Gastric Corpus. Gastroenterology 159, 2116–2129. 10.1053/j.gastro.2020.08.027 32835664 PMC7725914

[B4] Cannon J. P. Lee T. A. Bolanos J. T. Danziger L. H. (2005). Pathogenic Relevance of Lactobacillus: a Retrospective Review of over 200 Cases. Eur. J. Clin. Microbiol. Infect. Dis. 24, 31–40. 10.1007/s10096-004-1253-y 15599646

[B5] Chen W. Liu F. Ling Z. Tong X. Xiang C. (2012). Human Intestinal Lumen and Mucosa-Associated Microbiota in Patients with Colorectal Cancer. PLoS ONE 7, e39743. 10.1371/journal.pone.0039743 22761885 PMC3386193

[B6] Chen Y. R. Zhou L. Z. Fang S. T. Long H. Y. Chen J. Y. Zhang G. X. (2019). Isolation of Desulfovibrio Spp. From Human Gut Microbiota Using a Next‐generation Sequencing Directed Culture Method. Lett. Appl. Microbiol. 68, 553–561. 10.1111/lam.13149 30835854

[B7] Eun C. S. Kim B. K. Han D. S. Kim S. Y. Kim K. M. Choi B. Y. (2014). Differences in Gastric Mucosal Microbiota Profiling in Patients with Chronic Gastritis, Intestinal Metaplasia, and Gastric Cancer Using Pyrosequencing Methods. Helicobacter 19, 407–416. 10.1111/hel.12145 25052961

[B8] Fisher L. Fisher A. Smith P. N. (2020). *Helicobacter pylori* Related Diseases and Osteoporotic Fractures (Narrative Review). Jcm 9, 3253. 10.3390/jcm9103253 PMC7600664 33053671

[B9] Gupta A. Shah K. Oza M. J. Behl T. (2019). Reactivation of P53 Gene by MDM2 Inhibitors: A Novel Therapy for Cancer Treatment. Biomed. Pharmacother. 109, 484–492. 10.1016/j.biopha.2018.10.155 30551517

[B10] Iino C. Shimoyama T. Chinda D. Sakuraba H. Fukuda S. Nakaji S. (2019). Influence of *Helicobacter pylori* Infection and Atrophic Gastritis on the Gut Microbiota in a Japanese Population. Digestion 101 (4), 422–432. 10.1159/000500634 31394526

[B11] International Agency for Research on Cancer (2020). Estimated Number of Deaths in 2020, Worldwide, Both Sexes, All Ages. Available at: https://gco.iarc.fr/today/online-analysis-pie (Accessed May 21, 2021).

[B12] Jersey H. S. M. Helem F. R. Giovanny R. P. Luana de O. L. Letícia M. L. Carla M. F. P. (2018). Gastric Cancer Cell Lines Have Different MYC -Regulated Expression Patterns but Share a Common Core of Altered Genes. Can. J. Gastroenterol. Hepatol. 2018, 5804376. 30410872 10.1155/2018/5804376 PMC6206580

[B13] Kaakoush N. O. Holmes J. Octavia S. Man S. M. Zhang L. Castaño-Rodríguez N. (2010). Detection of Helicobacteraceae in Intestinal Biopsies of Children with Crohn's Disease. Helicobacter 15 (6), 549–557. 10.1111/j.1523-5378.2010.00792.x 21073612

[B14] Lanas A. Chan F. K. L. (2017). Peptic Ulcer Disease. The Lancet 390, 613–624. 10.1016/s0140-6736(16)32404-7 28242110

[B15] Li Y. Guo B. Wu Z. Wang W. Li C. Liu G. (2020). Effects of Fermented Soybean Meal Supplementation on the Growth Performance and Cecal Microbiota Community of Broiler Chickens. Animals 10 (6), 1098. 10.3390/ani10061098 PMC7341335 32630490

[B16] Li Y. Xia R. Zhang B. Li C. (2018). Chronic Atrophic Gastritis: A Review. J. Environ. Pathol. Toxicol. Oncol. 37, 241–259. 10.1615/jenvironpatholtoxicoloncol.2018026839 30317974

[B17] Li Y. Zhang Y. Meng H. Liao M. Su Z. Zhai M. (2019). Efficacy and Safety of Acupuncture Therapy for Chronic Atrophic Gastritis. Medicine (Baltimore) 98, e17003. 10.1097/md.0000000000017003 31464956 PMC6736333

[B18] Liu X. Mao B. Gu J. Wu J. Y. Cui S. M. Wang G. (2021). Blautia-a New Functional Genus with Potential Probiotic Properties? Gut microbes 13, 1–21. 10.1080/19490976.2021.1875796 PMC7872077 33525961

[B19] Lukic J. Strahinic I. Milenkovic M. Golic N. Kojic M. Topisirovic L. (2013). Interaction of Lactobacillus Fermentum BGHI14 with Rat Colonic Mucosa: Implications for Colitis Induction. Appl. Environ. Microbiol. 79, 5735–5744. 10.1128/aem.01807-13 23851097 PMC3754154

[B20] Man S. M. Zhang L. Day A. S. Leach S. Mitchell H. (2008). Detection of Enterohepatic and GastricHelicobacterSpecies in Fecal Specimens of Children with Crohn's Disease. Helicobacter 13, 234–238. 10.1111/j.1523-5378.2008.00607.x 18665930

[B21] Qin R. Wang J. Chao C. Yu J. Copeland L. Wang S. (2021). RS5 Produced More Butyric Acid through Regulating the Microbial Community of Human Gut Microbiota. J. Agric. Food Chem. 69, 3209–3218. 10.1021/acs.jafc.0c08187 33630575

[B22] Schenk R. L. Strasser A. Dewson G. (2017). BCL-2: Long and Winding Path from Discovery to Therapeutic Target. Biochem. Biophysical Res. Commun. 482 (3), 459–469. 10.1016/j.bbrc.2016.10.100 28212732

[B23] Si Y. C. Miao W. N. He J. Y. Chen L. Wang Y. L. Ding W. J. (2018). Regulating Gut Flora Dysbiosis in Obese Mice by Electroacupuncture. Am. J. Chin. Med. undefined, 1–17. 10.1142/S0192415X18500763 30284469

[B24] Smet A. Menard A. (2020). Review: Other Helicobacter Species. Helicobacter 24, e12744. 10.1111/hel.12645 32918348

[B25] Taniguchi K. Iwatsuki A. Sugito N. Shinohara H. Kuranaga Y. Oshikawa Y. (2018). Oncogene RNA Helicase DDX6 Promotes the Process of C-Myc Expression in Gastric Cancer Cells. Mol. Carcinogenesis 57 (5), 579–589. 10.1002/mc.22781 29314290

[B26] Tarasconi A. Coccolini F. Biffl W. L. Tomasoni M. Ansaloni L. Picetti E. (2020). Perforated and Bleeding Peptic Ulcer: WSES Guidelines. World J. Emerg. Surg. 15, 3. 10.1186/s13017-019-0283-9 31921329 PMC6947898

[B27] Tong-peng X. Pei M. Wen-yu W. You S. Yan-fen W. Tao Y. (2019). KLF5 and MYC Modulated LINC00346 Contributes to Gastric Cancer Progression through Acting as a Competing Endogeous RNA and Indicates Poor Outcome. Cel Death Differ. 26 (11), 2179–2193. 10.1038/s41418-018-0236-y PMC6888833 30770877

[B28] Wang J. Zhang C. Guo C. Li X. (2019). Chitosan Ameliorates DSS-Induced Ulcerative Colitis Mice by Enhancing Intestinal Barrier Function and Improving Microflora. Int. J. Mol. Sci. 20 (22), 5751. 10.3390/ijms20225751 PMC6888260 31731793

[B29] Wang L. Zhou J. Xin Y. Geng C. Tian Z. Yu X. (2016). Bacterial Overgrowth and Diversification of Microbiota in Gastric Cancer. Eur. J. Gastroenterol. Hepatol. 28, 261–266. 10.1097/meg.0000000000000542 26657453 PMC4739309

[B30] Waters J. L. Ley R. E. (2019). The Human Gut Bacteria Christensenellaceae Are Widespread, Heritable, and Associated with Health. BMC Biol. 17, 83. 10.1186/s12915-019-0699-4 31660948 PMC6819567

[B31] Won K. Y. Kim G. Y. Kim H. K. Song M. J. Choi S. I. Bae G. E. (2019). The Expression of C-MYC in Gastric Adenocarcinoma Is Associated with PD-L1 and FOXP3 Expression: C-MYC Overexpression Is a Good Prognostic Factor. Pathol. - Res. Pract. 215 (11), 152639. 10.1016/j.prp.2019.152639 31582185

[B32] Yoldemir E. A. Ozturk G. Z. Akarsu M. Ozcan M. (2021). Is There a Correlation between Hypomagnesemia Linked to Long-Term Proton Pump Inhibitor Use and the Active Agent? Wiener klinische Wochenschrift, 7634. 10.1007/s00508-021-01834-x 33751184

[B33] Yu C. Su Z. Li Y. Li Y. Liu K. Chu F. (2020). Dysbiosis of Gut Microbiota Is Associated with Gastric Carcinogenesis in Rats. Biomed. Pharmacother. 126, 110036. 10.1016/j.biopha.2020.110036 32172061

[B34] Zhang J. Huang K. Zhong G. Huang Y. Li S. Qu S. (2016). Acupuncture Decreases NF-Κb P65, miR-155, and miR-21 and Increases miR-146a Expression in Chronic Atrophic Gastritis Rats. Evid. Based Complement. Alternat Med. 2016, 9404629. 10.1155/2016/9404629 27293468 PMC4887647

[B35] Zhang Z. Zhu L. Ma Y. Wang B. Ci C. Zhang J. (2021). Study on the Characteristics of Intestinal Flora Composition in Gastric Cancer Patients and Healthy People in the Qinghai-Tibet Plateau. Appl. Biochem. Biotechnol.. 10.1007/s12010-021-03732-4 PMC9007807 34792749

[B36] Zhao X. Wu M. Zhang D. Sun Y. Yang Y. Xie H. (2018). The Relationship of Interpersonal Sensitivity and Depression Among Patients with Chronic Atrophic Gastritis: The Mediating Role of Coping Styles. J. Clin. Nurs. 27, e984–e991. 10.1111/jocn.14114 29052273

[B37] Zhu M. P. Zhu B. X. (2018). The Research of Helicobacter Species on Ulcerative Colitis. Chin. J. Gastroenterol. Hepatol. 27 (02), 126–129.

